# Nomogram prediction of the lymph-vascular space invasion in cervical cancer: comparison of 2009 and 2018 staging systems

**DOI:** 10.3389/fonc.2025.1505512

**Published:** 2025-03-06

**Authors:** Suyu Li, Yusha Chen, Xizhen Huang, Xiaoying Chen, Xiaoyang Li, Guangrun Zhou, Liyuan Huang, Qiuyuan Huang, Lingsi Chen, Zhonghang Xie, Xiangqin Zheng

**Affiliations:** ^1^ Department of Radiation Oncology, College of Clinical Medicine for Obstetrics & Gynecology and Pediatrics, Fujian Medical University, Fujian Maternity and Child Health Hospital, Fuzhou, China; ^2^ Cervical Disease Diagnosis and Treatment Health Center, Fujian Maternity and Child Health Hospital, College of Clinical Medicine for Obstetrics & Gynecology and Pediatrics, Fujian Medical University, Fuzhou, China; ^3^ Department of Radiation Oncology, Fujian Children’s Hospital (Fujian Branch of Shanghai Children’s Medical Center), College of Clinical Medicine for Obstetrics & Gynecology and Pediatrics, Fujian Medical University, Fuzhou, China; ^4^ Department of Gynecology, College of Clinical Medicine for Obstetrics & Gynecology and Pediatrics, Fujian Medical University, Fujian Maternity and Child Health Hospital, Fuzhou, China; ^5^ Department of Emergency Response, Fujian Provincial Center for Disease Control and Prevention, Fuzhou, Fujian, China

**Keywords:** cervical cancer, nomogram, lymph-vascular space invasion, FIGO stage, gynecological malignant tumor, early-stage

## Abstract

**Background:**

Lymph-vascular space invasion (LVSI) is a crucial prognostic factor in cervical cancer (CC), and its assessment is essential for developing personalized treatment strategies.

**Objective:**

The primary objective of this study was to focused on constructing LVSI predictive model based on clinical indicators and evaluating its predictive performance across different FIGO staging cohorts.

**Study design:**

We included 691 patients, with 348 patients having 2009 FIGO stage IB1-IIA2 CC assigned to Cohort 1, and 343 patients with 2018 FIGO stage IB1-IIIC1r CC assigned to Cohort 2. In Cohort 1, univariable and multivariable regression analyses, along with Mallows’ Cp, R squared-R, and LASSO, were used to select variables forming Model 1. Model 2 included the FIGO stage. We compared the contribution of different FIGO stages to the LVSI prediction model in both cohorts. The final LVSI prediction model for the entire cohort was constructed using selected variables and risk stratification was established. The models were evaluated through internal validations using ROC curves, C-index, Clinical Impact Curve (CIC), and Decision Curve.

**Results:**

Five variables were incorporated into Model 1: age, Pathology, Depth of Stromal Invasion (DSI), SCC-Ag, and Lactate Dehydrogenase (LDH). Model 2 was established by incorporating the FIGO staging system. Compared with the two models, there was no significant difference in ROC, ΔC-index and ΔNRI. Adding FIGO clinical staging did not significantly improve predictive value. Model 1’s variable were included in the nomogram for the combined cohort. The AUC for the model-development cohort and validation cohort was 0.754 (95% CI: 0.711, 0.798) and 0.789 (95% CI: 0.727, 0.852), respectively. In both cohorts, risk stratification effectively distinguished the high-risk group, which had a significantly higher proportion of positive cases compared to the low and middle-risk groups (p < 0.01).

**Conclusion:**

Our nomogram predictive model demonstrates robust LVSI prediction performance across different staging systems.

## Introduction

Cervical cancer (CC) is the most common malignant tumor of the female reproductive system and the fourth most common malignancy in women worldwide (1). In 2020, China reported approximately 111,820 new cases of cervical cancer (CC) and 61,579 CC-related deaths (1). The incidence is rising and affecting younger women, with 51.1% of HPV-related cervical cancer cases occurring in women aged 40-54 (2). The estimated number of new invasive cervical cancer cases in the United States in 2024 is 13,820 and 4,360 deaths (3). With the widespread implementation of cervical cancer screening, the detection rate of early-stage cervical cancer has increased (4). The incidence of squamous cell carcinoma (SCC) remains high, fluctuating between 75% and 90%, while the frequency of adenocarcinoma is on the rise (5). Due to the delay of childbearing, the requirement of early cervical cancer to retain fertility function has become more critical. Lymphovascular space invasion (LVSI) refers to the presence of clusters of cancer cells within the lumen of blood vessels or lymphatics. LVSI status is a significant prognostic factor for recurrence in early-stage CC following fertility-sparing treatment (6). In IB1 stage tumors, the presence of LVSI is considered a contraindication for fertility preservation (7).

Staging is a crucial prognostic factor, early-stage CC has a relatively high five-year survival rate. However, there remains a recurrence rate of 10%-30% (7). The incidence of LVSI in early-stage cervical cancer varies significantly. In IB1 cervical cancer, the reported incidence is 27.1% (8). However, another study involving 347 cases of stage IB-IIA cervical cancer found that the incidence of LVSI could be as high as 51.8% (9). In 1990, the first prospective study (GOG-49) involving 645 cases of FIGO stage I CC found that the 3-year disease-free survival rate was significantly associated with tumor size, depth of stromal invasion (DSI), and the presence of lymph-vascular invasion (LVSI) (p=0.006) (10). Sedlis conducted one of the largest prospective, multicenter, randomized phase III trials (GOG#92), involving 277 patients with early-stage cervical cancer. The study assessed several risk factors, including lymphovascular space invasion (LVSI), which was present in 70.3% of the patients. The recurrence rate in the radiotherapy group was only 12%, compared to 21% in the control group, with an adjusted 44% reduction in the risk of recurrence (p=0.019) (11). Recently, several other authors have also identified LVSI as a prognostic factor for early cervical lesions (12–14). The NCCN guidelines now include LVSI, along with depth of stromal invasion (DSI) and tumor size as one of the risk factors for considering adjuvant radiotherapy in early-stage cervical cancer (15). Consequently, the evaluation of LVSI is essential to provide valuable clinical guidance.

Nowadays, nomogram models established by combining clinical indicators have proved to be reliable and practical for the risk and prognosis stratification of LVSI. These models facilitate comparisons between different predictors, allowing for the identification of more valuable predictors and the extension of their usefulness. Due to the FIGO clinical staging system was revised in 2018 to include lymph node metastasis (LNM) and tumor size. With the application of the FIGO imaging staging system, it is crucial to evaluate whether the LVSI predictive model developed under the 2009 FIGO clinical staging system can be extended to the 2018 FIGO staging system and whether they remain clinically practical across different risk categories.

Therefore, we conducted a retrospective analysis of CC with consecutive diagnoses of 2009 FIGO stage IB1-IIA2 and 2018 FIGO stage IB1-IIICr. These patients underwent radical hysterectomy and pelvic ± abdominal para-aortic lymphadenectomy (RHPL), with postoperative pathology indicating the presence or absence of LVSI. Our objective was to incorporate the corresponding FIGO stages into different cohorts and to determine the predictive value of various staging systems for LVSI, as well as the feasibility of expanding the predictive model.

## Materials and methods

### Study participants

**Figure 1:**
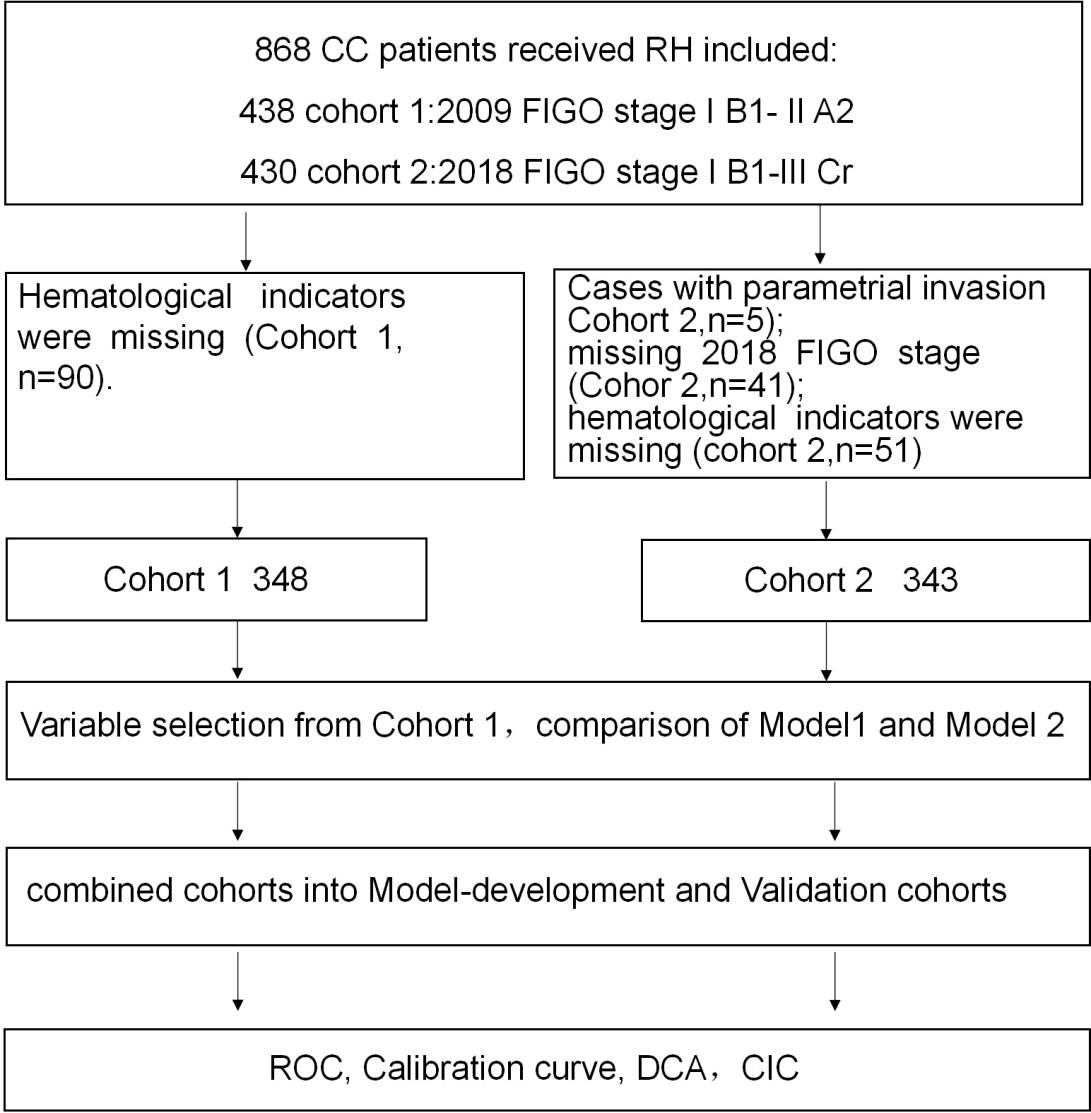
Study design and flowchart. ROC, Receiver Operating Characteristic; DCA, Decision Curve Analysis, CIC, Clinical Impact Curve, NRI, Net Reclassification Improvement, IDI, Integrated Discrimination Improvement.

A total of 868 patients who underwent surgical treatment at Fujian Maternity and Child Health Hospital were included in this study. This comprised 438 patients with pathologically-proven 2009 FIGO stage IB1-IIA2 cervical cancer (cohort 1) and 430 patients with pathologically-proven 2018 FIGO stage IB1-IIICr cervical cancer (cohort 2). The inclusion criteria were as follows: (1) Cohort 1: Patients diagnosed according to the 2009 FIGO clinical staging system (stage IB1-IIA2) and who underwent radical hysterectomy by the same group of surgeons between February 2011 and December 2018. (2) Cohort 2: Patients diagnosed according to the 2018 FIGO imaging staging system (stage IB1-IIICr) and who underwent radical hysterectomy by two groups of surgeons between January 2019 and December 2023. (3) Availability of complete clinical data, including LVSI. A total of 110 patients were excluded due to the following factors: (1) Preoperative diagnosis of parametrial invasion in stage IIB cases (cohort 2, n=5). (2) Missing data for the 2018 FIGO imaging staging system (cohort 2, n=41). (3) Missing complete blood count or tumor marker data (cohort 1, n=90; cohort 2, n=51). The final study population consisted of 348 patients staged according to the 2009 FIGO system and 343 patients staged according to the 2018 FIGO system who met the eligibility criteria (**Figure 1**).

### Data collection

Clinical information, including patient FIGO stage, age, tumor size, and preoperative hematological data, was collected for all patients. Pathological features mainly included LVSI, pathology and DSI. Based on the positive or negative of LVSI determined by surgical pathology, patients were categorized into two groups. Hematological data were collected one week before treatment and included white blood cell (WBC) count, neutrophil percentage (NE%), lymphocyte percentage (LY%), platelet count (PLT), lactate dehydrogenase (LDH), squamous cell carcinoma antigen (SCC-Ag), cancer antigen 125 (CA125) and cancer antigen 19-9 (CA19-9). We calculated the neutrophil-to-lymphocyte ratio to generate the NLR. FIGO stage, tumor size, LVSI, DSI, SCC-Ag, CA125 and CA19-9 were considered categorical variables, while age, WBC, NE, LY, PLT, LDH, and NLR were treated as continuous variables. Missing hematological indices account for 10% of the total sample size. We used decision tree imputation to supplement the missing variables (16), ensuring data integrity, and performed data analysis using R package software. The Transparent Reporting of a multivariable prediction model for Individual Prognosis Or Diagnosis (TRIPOD) guidelines were consulted to structure this observational cohort study (17).

### Statistical analysis

We utilized the chi-square test probability method to compare the classification variables. Pearson’s chi-squared test was employed to analyze categorical variables across various risk groups. Quartile ranges (Q1-Q3) were utilized to represent data in skewed distributions, while mean values ± standard deviations were compared for variables with a normal distribution. Nomogram prediction models were established following three steps. Firstly, a univariate and multivariate regression analysis was conducted to identify features significantly related to LVSI. Secondly, in Cohort1, we employed three advanced statistical methods —Mallows Cp, R squared-R, and Least Absolute Shrinkage and Selection Operator (LASSO)—to select variables. Variable selection criteria for Mallows Cp and R squared-R were determined by the Bayesian information criterion (BIC). Cross-validation was employed to determine suitable tuning parameters (λ) for LASSO logistic regression, with the most significant features selected by LASSO. Based on the results from Mallows Cp, R squared-R, and LASSO, variables were incorporated into the Model 1. In Model 2, the FIGO variable was included, and the predictive value for LVSI was compared between the two cohorts. The models were evaluated using receiver operating characteristic (ROC) curves, Harrell’s concordance index (C-index), net reclassification index (NRI) and integrated discrimination improvement (IDI). Thirdly, based on the above analysis results, five variables from Model 1 were selected to construct a predictive model for the entire combined cohort. This model underwent internal validation. Ultimately, the predictive model is applicable to both the 2009 FIGO staging system and the 2018 FIGO staging system. The data analysis in this study was conducted using the R statistical software package (http://www.R-project.org, The R Foundation) and Free Statistics software version 1.9. A two-tailed test was employed to determine the significance of the results, with a p-value of less than 0.05 considered statistically significant.

## Results

### Clinical characteristics

A total of 691 patients were included in this study. Cohort 1, used for developing the model, comprised 348 patients with pathologically confirmed 2009 FIGO stage IB1-IIA2 cervical cancer (CC). Cohort 2 included 343 patients with pathologically confirmed 2018 FIGO stage IB1-IIICr cervical cancer (CC). The characteristics were shown in **Supplementary Table S1**. In cohort 1, the LVSI frequencies for Cohort 1 and Cohort 2 were 32.5% and 49.3%, respectively (p < 0.001). There were statistically significant differences between the two groups in terms of age, FIGO, tumor size, pelvic lymph node, and NE. However, there were no statistically significant differences in pathology and hematological markers WBC, PLT, LY, LDH, NLR, SCC-Ag, CA199, CA125.

### Univariate and multivariate analysis for LVSI

The univariate analysis indicated that DSI, tumor size, pathology, SCC-Ag and LDH were all associated with LVSI. Multivariate analysis confirmed that DSI, age, pathology, and SCC-Ag are independent factors for LVSI. The results of univariate and multivariate logistic regression analyses are presented in **Supplementary Tables S2**.

### Variable selection

**Figure 2:**
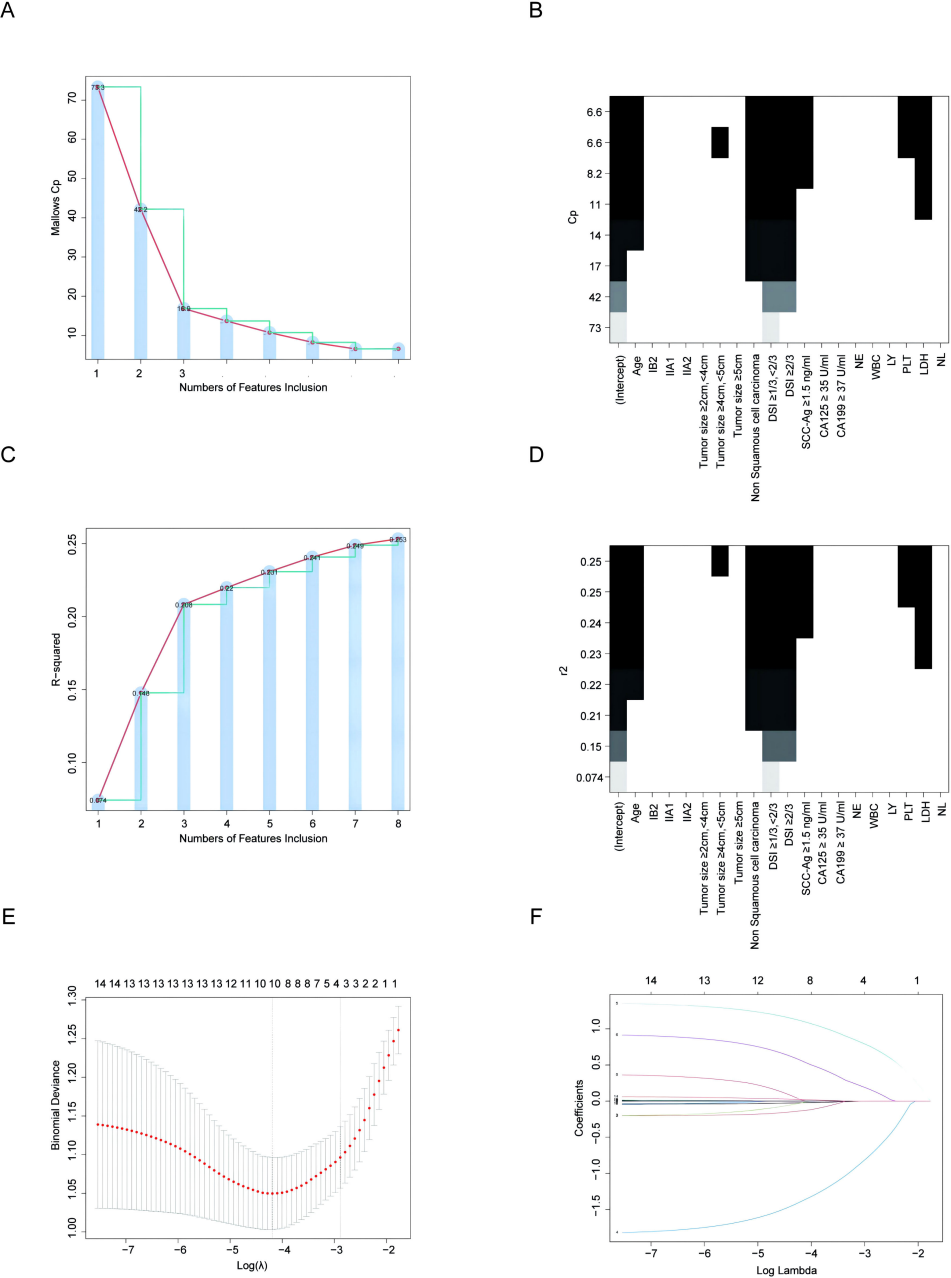
Methods of variable selection. **(A, B)** Variable selection was performed using the Mallows Cp method. **(A)** The minimum Mallows Cp value of 6.6 was identified at the inflection point of the segmented line. **(B)** The y-axis represents the Mallows Cp values, while the x-axis denotes the variables. **(C, D)** Variable selection was performed using the R squared-R method. **(C)** The maximum R squared-R of 0.253 was identified at the inflection point of the segmented line. **(D)** The y-axis represents the R squared-R values, and the x-axis denotes the variables. **(E)** Cross-validation was utilized to select the tuning parameter (λ). **(F)** LASSO coefficient curve for LVSI-related variables in cohort 1. LASSO, least absolute shrinkage and selection operator; FIGO, the International Federation of Gynecology and Obstetrics; DSI, depth of stromal invasion; SCC-Ag, squamous cell carcinoma antigen; CA125, cancer antigen 125; CA199, cancer antigen 199; WBC, white blood cell; NE, neutrophil; LY, lymphocyte; PLT, platelet; LDH, lactate dehydrogenase; NLR, neutrophil-to-lymphocyte ratio.

Three common methods—Mallows Cp, R squared-R and Least Absolute Shrinkage and Selection Operator (LASSO)—were used to select variables. Mallows Cp yielded eight variables: age, tumor size, DSI, pathology, PLT, SCC-Ag and LDH. The minimum Mallows Cp value was 6.6 (**Figures 2A, B**). R squared-R yielded eight variables: age, DSI, tumor size, pathology, SCC-Ag, PLT and LDH. The maximum R squared-R was 0.253 (**Figures 2C, D**). All selected variables had significant statistical differences (all P < 0.05). LASSO identified five statistically significant variables: age, DSI, pathology, SCC-Ag, and LDH (lambda value = 0.03). As shown in **Figures 2E, F**, the coefficient profile plot was produced against the sequence of ln(λ).

### Development and comparison of Model 1 and Model 2

**Figure 3:**
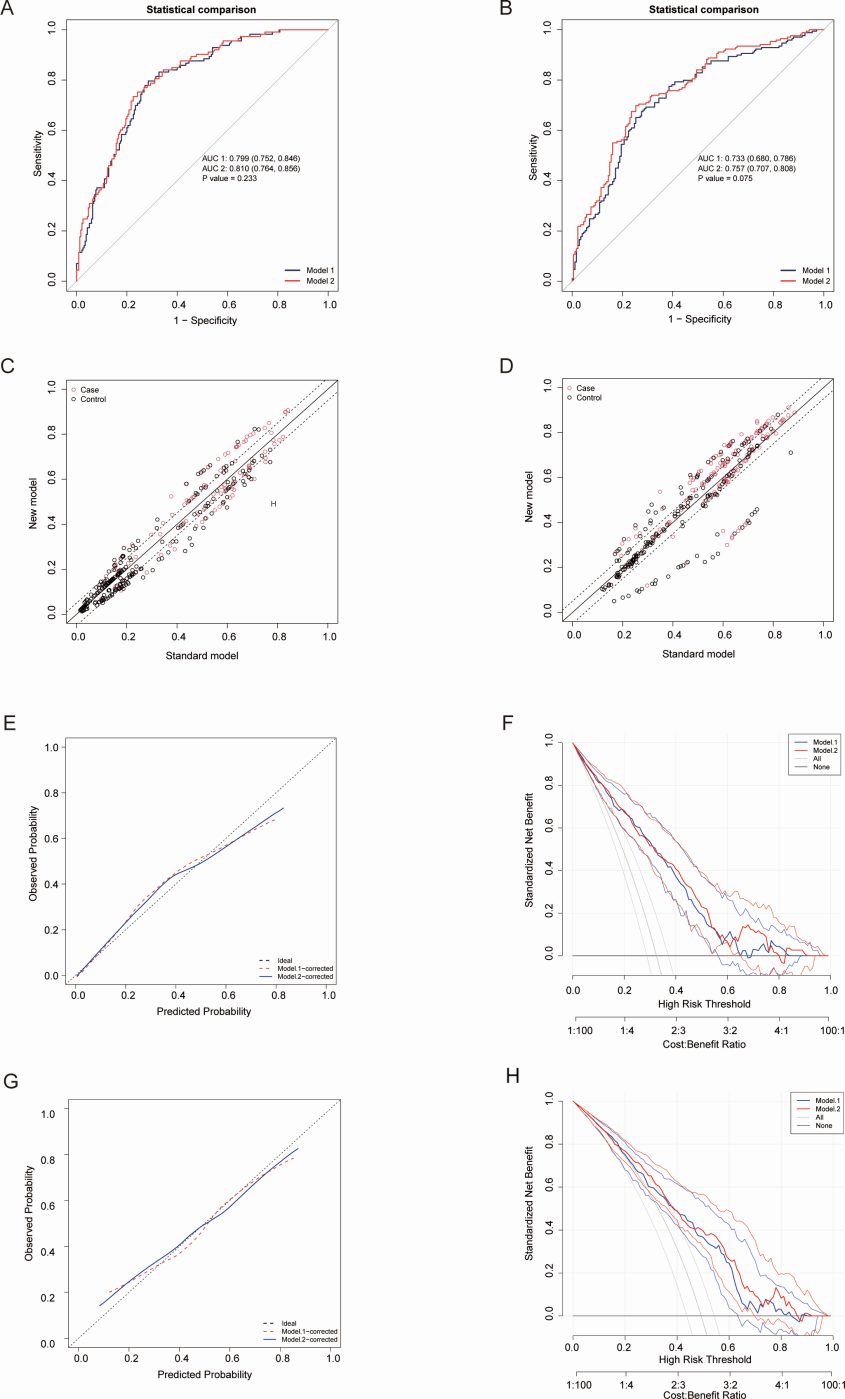
Comparison of Model 1 and Model 2 using ROC, NRI.diff, calibration curve and DCA. **(A)** ROC for the model 1 and model 2 in Cohort 1. **(B)** ROC for the model 1 and model 2 in Cohort 2. **(C)** difference in Net Reclassification Improvement (NRI.diff) for the model 1 and model 2 in Cohort 1. **(D)** NRI.diff for the model 1 and model 2 in Cohort 2. **(E)** Calibration curves for Cohort 1 between two models. **(F)** DCA plots for Cohort 1 between two models. **(G)** Calibration curves for Cohort 2 between two models. **(H)** DCA plots for Cohort 2 between two models. ROC, Receiver Operating Characteristic. NRI.diff, difference in Net Reclassification Improvement. DCA, Decision Curve Analysis.

Based on the results of the univariate and multivariate regression analyses, as well as the Mallows Cp, R squared-R and LASSO methods, five variables were incorporated into Model 1: age, DSI, pathology, SCC-Ag, and LDH. FIGO stage was added to establish Model 2. ROC curve, Net Reclassification Improvement difference, Calibrate and Decision curve were employed to assess the clinical utility of two models in Cohort 1 and Cohort 2 respectively (**Figure 3**). C-index, NRI and IDI metrics were used to evaluate the efficiency of the models (**Tables 1**, **2**). In Cohort 1, Model 1: AUC= 0.799 (95% CI: 0.752, 0.846) VS Model 2: AUC= 0.810 (95% CI: 0.764, 0.856) (P = 0.233). ΔC-index (P = 0.233), NRI [0.0383, 95% CI: -0.0384, 0.115] (P < 0.3277), and IDI [0.0197, 95% CI: 0.0039, 0.0355](P < 0.015). In Cohort 2, Model 1: AUC= 0.733 (95% CI: 0.680, 0.786) VS Model 2: AUC= 0.757 (95% CI: 0.707, 0.808) (P = 0.075). ΔC-index (P = 0.075), NRI [0.0451, 95% CI:-0.036, 0.1263] (P < 0.275), and IDI [0.0373, 95% CI:0.0168, 0.0579](P < 0.01).

**Table 1:** Effectiveness of model 1 and model 2 in predicting LVSI in cohort 1.

	C-Index [95% CI]	ΔC-index (P-value)	NRI [95% CI]	ΔNRI (P-value)	IDI [95% CI]	ΔIDI (P-value)
model1	0.799 (0.752, 0.846)	–				
model2	0.810 (0.764, 0.856)	0.233	0.0383 [-0.0384 0.115]	0.3277	0.0197 [0.0039 0.0355]	0.01457

NRI, Net Reclassification Improvement, IDI, Integrated Discrimination Improvement.

**Table 2:** Effectiveness of model 1 and model 2 in predicting LVSI in cohort 2.

	C-index [95% CI]	ΔC-index (P-value)	NRI [95% CI]	ΔNRI (P-value)	IDI [95% CI]	ΔIDI (P-value)
model1	0.733 (0.680, 0.786)	–				
model2	0.757 (0.707, 0.808)	0.075	0.0451 [-0.036 0.1263]	0.27571	0.0373 [0.0168 0.0579]	0.00037

NRI, Net Reclassification Improvement, IDI, Integrated Discrimination Improvement

### Confirmation and validation of the nomogram Model 1 in the overall cohort

**Figure 4:**
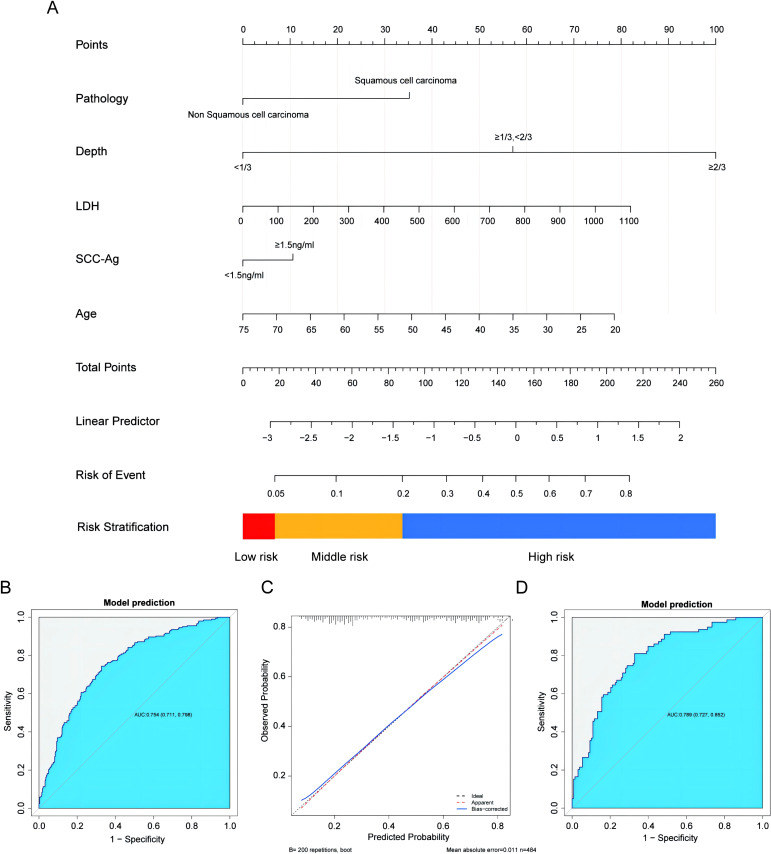
The nomogram, ROC, calibration curve predicting LVSI using Model-developed Cohort. **(A)** A Linear Predictor of -2.567 was assigned a risk value of 0.05 and was used as the threshold to differentiate between low and medium risk for LNM. A Linear Predictor of -1.386 was assigned a risk value of 0.20 and was used as the threshold to differentiate between medium and high risk. **(B)** ROC for the Model-development Cohort. **(C)** Calibration curve for the Model-development Cohort. The observed results of the model closely align with the ideal predicted outcomes. **(D)** ROC for the Validation Cohort. ROC, Receiver Operating Characteristic.

**Figure 5:**
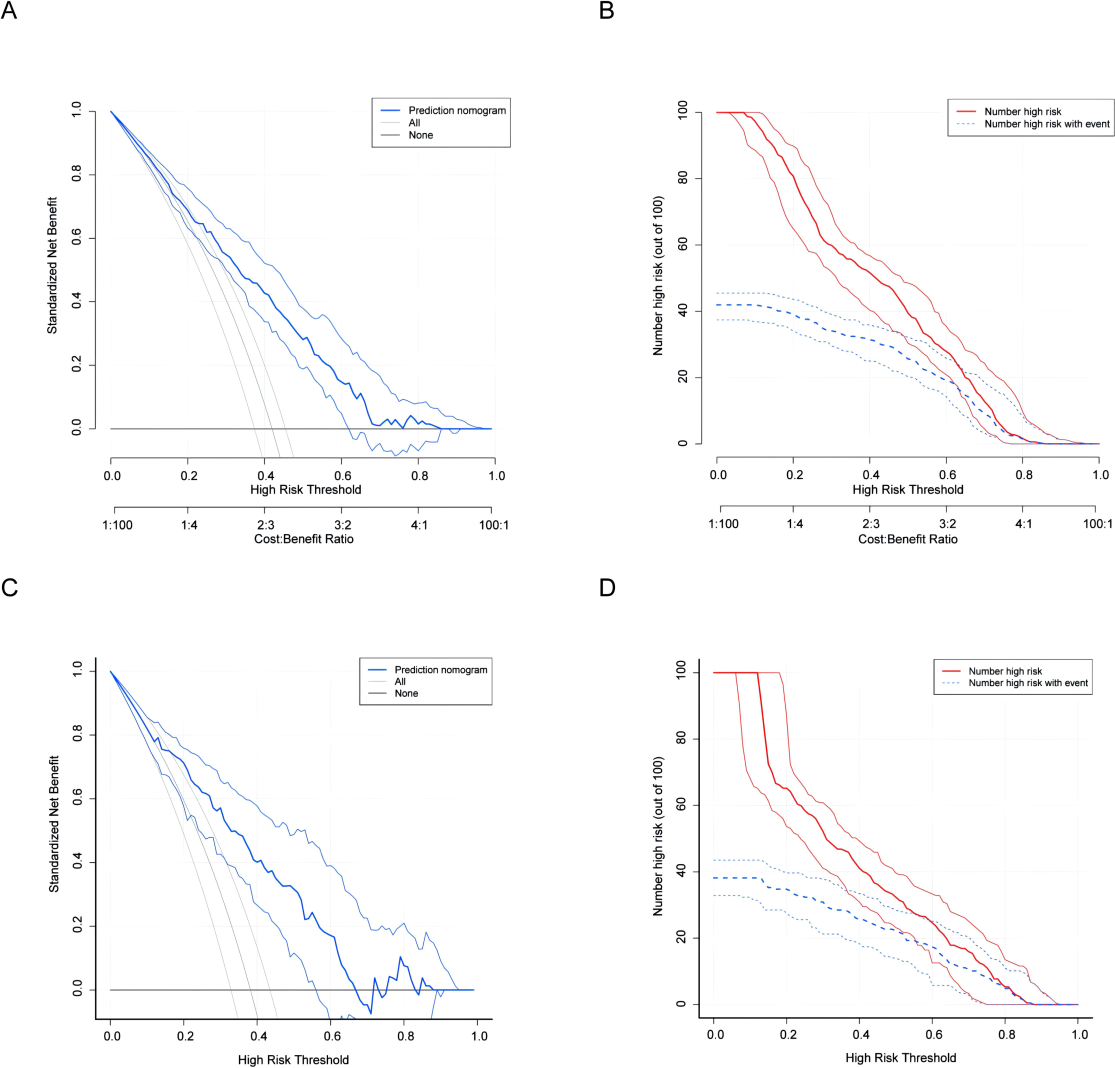
DCA and CIC plots across Model-development and Validation Cohort. DCA and CIC curves illustrate the predictive performance and clinical utility comparison between Model-development and Validation Cohort: **(A)** DCA curve for Model-development Cohort. **(B)** CIC curve for Model-development Cohort. **(C)** DCA curve for Validation Cohort. **(D)** CIC curve for Validation Cohort. CIC demonstrates that the true positive rate (blue line) and the false positive rate (red line) converge at different risk thresholds, indicating a significant increase in net benefit. DCA, Decision Curve Analysis. CIC, Clinical Impact Curve.

We confirmed the significance of the five variables: age, DSI, pathology, SCC-Ag, and LDH from Model 1 within the overall combined cohort. The characteristics of model-development and validation Cohorts were showed in **Table 3**. These variables were incorporated into a nomogram prediction model to enhance its predictive accuracy and clinical utility (**Figure 4A**). The details of the model validation process and the performance metrics are discussed below. In the model-development Cohort, the model’s discrimination accuracy was 0.754 (95% CI: 0.711, 0.798; **Figure 4B**). The Hosmer-Lemeshow test showed a satisfactory fit for the model (R2 = 0.249, X_2_ = 2.315, P = 0.97). A well-calibrated prediction model would closely fit the dashed line (**Figure 4C**). Brier Scores closer to zero show better calibration. Internal validation results yielded an AUC 0.789 (95% CI: 0.727, 0.852; Brier = 0.186, X_2_ = 21.508, P = 0.006) (**Figure 4D**). DCA and Clinical Impact Curve (CIC) results were shown in (**Figure 5**).

**Table 3:** Characteristics of variables included in the model-development and validation cohorts.

Characteristics	Total (n = 691)	The model-development Cohort (n = 484)	The validation Cohort (n = 207)	p
age, Mean ± SD	48.5 ± 9.3	48.3 ± 9.4	48.8 ± 9.0	0.576
pathology, n (%)				0.242
Squamous cell carcinoma	524 (75.8)	361 (74.6)	163 (78.7)	
Non Squamous cell carcinoma	167 (24.2)	123 (25.4)	44 (21.3)	
DSI, n (%)				0.067
<1/3	251 (36.3)	189 (39)	62 (30)	
≥1/3,<2/3	219 (31.7)	149 (30.8)	70 (33.8)	
≥2/3	221 (32.0)	146 (30.2)	75 (36.2)	
SCC-Ag (ng/ml), n (%)				0.478
<1.5	318 (46.0)	227 (46.9)	91 (44)	
≥1.5	373 (54.0)	257 (53.1)	116 (56)	
LDH, Median (IQR)	165.1 (143.0, 196.6)	165.0 (143.1, 196.0)	166.0 (142.7, 201.0)	0.639
LVSI, n (%)				0.355
Negative	409 (59.2)	281 (58.1)	128 (61.8)	
Positive	282 (40.8)	203 (41.9)	79 (38.2)	

FIGO, the International Federation of Gynecology and Obstetrics; DSI, depth of stromal invasion; LVSI, Lymph-vascular invasion; SCC-Ag, squamous cell carcinoma antigen; CA125, Cancer Antigen 125; CA199, Cancer Antigen 199; WBC, White Blood Cell; NE, neutrophil; LY, lymphocyte; PLT, Platelet; LDH, Lactate Dehydrogenase; NLR, neutrophil-to-lymphocyte ratio.

### LVSI risk groups

Using the linear predictors, a predicted probability of LVSI < 5% was defined as low risk, 5%-20% as middle risk, and > 20% as high risk. The distribution of risk groups and associated outcomes in both the model-development cohort (484 patients) and the validation cohort (206 patients) are presented in **Table 4**. In the model-development cohort, the low-risk group (1.9% of patients) had 3 negative cases and no positive cases. The middle-risk group (21.5% of patients) had 93 negative and 11 positive cases, while the high-risk group (76.7% of patients) had 189 negative and 182 positive cases, with Fisher’s exact test showing a significant association (p < 0.001). Similarly, in the validation cohort, the low-risk group (1.5% of patients) had 3 negative cases and no positive cases. The middle-risk group (18.4% of patients) had 30 negative and 8 positive cases, and the high-risk group (80.1% of patients) had 84 negative and 81 positive cases, with a significant association (p < 0.002) (**Table 4**).

**Table 4:** LVSI risk groups in the model-development and validation cohorts.

Characteristics	The model-deveolpment Cohort			The validation Cohort		
	Total (484)	Negtive (291)	Positive (193)	Total (206)	Negtive (117)	Positive (89)
low risk group	9 ( 1.9)	9 (3.1)	0 (0)	3 ( 1.5)	3 (2.6)	0 (0)
middle risk group	104 (21.5)	93 (32)	11 (5.7)	38 (18.4)	30 (25.6)	8 (9)
high risk group	371 (76.7)	189 (64.9)	182 (94.3)	165 (80.1)	84 (71.8)	81 (91)
Statistic (Fisher)	p< 0.001			p< 0.002		

### Comment

#### Principal findings

The primary results of this study indicate that the LVSI prediction model variables, identified using the 2009 FIGO staging Cohort 1, have maintained robust predictive performance within the 2018 FIGO staging system Cohort 2. The inclusion of the FIGO staging system did not significantly enhance the accuracy of LVSI prediction. Therefore, a combined cohort comprising the 2009 FIGO Cohort 1 and the 2018 FIGO Cohort 2 was used to construct the LVSI prediction model. For the overall cohort, a nomogram was built based on DSI, SCC, age, LDH, and pathology, and applied to the model-development and validation cohorts. In the model-development Cohort, the model’s discrimination accuracy was 0.754 (95% CI: 0.711, 0.798). The validation results yielded an AUC of 0.789 (95% CI: 0.727, 0.852). These findings confirm the robustness and clinical utility of LVSI nomogram prediction model developed from the combined FIGO 2009 and 2018 cohorts. The model’s strong predictive performance and calibration across different cohorts highlight its potential for accurate individualized patient management and risk assessment in clinical practice. The stratification into low, middle, and high-risk categories based on predicted probabilities further enhances its practical application, allowing for more tailored and effective patient care.

#### Results in the context of what is known

Among the risk factors included in the nomogram prediction model, DSI (Depth of Stromal Invasion) emerged as the most effective predictor of LVSI. Preoperative MRI imaging and cervical cone biopsy pathology can objectively and accurately determine the extent of DSI. Additionally, pathological slides facilitate precise repeated measurements. Wei Du et al’s model for predicting LVSI before surgery also confirmed the important predictive value of DSI, which is consistent with our study (18). Transvaginal or rectal ultrasound (TRUS/TVUS) and conventional MRI had a high diagnostic accuracy for DSI, 93% and 88%, respectively. The mean difference between pathological findings and MRI in measuring the maximum DSI was -0.65mm (95% limits of agreement: -9.37mm to 8.07mm) (19). When the DSI can be assessed with high accuracy, preoperative prediction of LVSI using the model becomes more feasible.

SCC-Ag was an independent risk factor for LVSI (P < 0.05) and was included in the prediction model, which was in accordance with Guo et al. (20) Our nomogram also highlight the higher propensity for LVSI in squamous cell carcinoma (SCC) compared to non SCC. Multiple studies have consistently demonstrated that the incidence of LVSI in SCC of the cervix is significantly higher than in adenocarcinoma (AC). In a retrospective analysis of 810 patients with stage IB-IIA cervical cancer (682 SCC and 128 AC), Xiuzhen Xie et al. found that the incidence of LVSI in SCC patients was significantly higher than in AC patients (23.90% vs. 8.59%, P < 0.05) (21). Similarly, a study by the GOG compared 645 SCC and 104 AC patients, revealing LVSI rates of 43% and 27%, respectively. This study also noted that AC patients had a significantly higher proportion of highly differentiated cells compared to SCC patients (42% vs. 14%) (22). Another research Lee et al. involving stage IB-IIA cervical cancer patients (636 SCC and 139 AC) also found a higher incidence of LVSI in SCC patients (27.3% vs. 10.8%), though the differentiation rates between the SCC and AC were similar (23).

Notably, this is the first known report of LDH and age being used in LVSI nomogram prediction model. we observed a significant increase in the LDH associated with an elevated risk of LVSI (24). LDH is a cellular enzyme that catalyzes the conversion of pyruvate to lactate under anaerobic conditions, serving as the rate-limiting step in this process (25). Tumor cells, due to their rapid growth and abnormal angiogenesis, often experience hypoxia, especially in the presence of LVSI, which may exacerbate hypoxia. NF-κB regulates the growth of tumor cells in a hypoxic microenvironment through competitive inhibition of PHD-2 mediated by pyruvate (26). Therefore, LVSI and LNM may be associated with elevated LDH levels (27). In predictive models for LNM in early-stage cervical cancer, age has been reported to be negatively associated with LNM (28–30). Moreover, there was a strong positive correlation between LVSI and LNM (8). LVSI positivity is frequently observed in patients with lymph node metastasis. The incidence of LVSI was higher in younger patients, with studies reporting an incidence of 50% in patients under 35 years compared to 39% in those over 35 years (31). Consistent with these predictive models, our model also demonstrates an inverse relationship between age and LVSI.

#### Clinical and research implications

This study confirmed that patients with a tumor size ≤2 cm, SCC < 1.5 ng/ml, and normal LDH levels have a very low risk of LVSI. At the same time, preoperative imaging features and pathological characteristics from cervical conization or extensive cervical resection provide relatively predictive determination of DSI risk factors, overcoming the bottleneck of preoperative LVSI prediction. LVSI-negative Patients with early-stage cervical cancer may benefit from fertility-sparing trachelectomy. When the tumor is larger than 2 cm, the recurrence rate is increased, especially the choice of minimally invasive or vaginal surgery recurrence rate is significantly higher than open surgery (32). Due to the high positive predictive value of sentinel node biopsy (33), the guidelines recommend sentinel node biopsy performed intraoperatively as well (34). Additionally, as we know neoadjuvant chemotherapy (NACT) reduces tumor volume preoperatively and increases the resection rate. Several studies have demonstrated its ability to improve the prognosis of cervical cancer (35). The efficacy of NACT remains controversial. A study has shown that NACT can reduce the detection rate of LVSI in CC. However, multiple intermediate-risk factors remain independent prognostic factors for recurrence and mortality, and postoperative adjuvant therapy is still necessary (36). Therefore, pre-NACT assessment of LVSI is highly valuable. Our model can achieve good prediction performance for both 2009 FIGO Cohort and 2018 FIGO Cohort. This simple nomogram histogram helps clinicians predict LVSI in early stage CC patients, which can optimize clinical treatment strategies.

Furthermore, numerous studies have explored the performance of deep learning models that combine clinical information and radiomic features in predicting LVSI in cervical cancer, with reported AUCs ranging from 0.659 to 0.91 (37–40). The preoperative radiomics prediction model, constructed using three radiomic features from T1 CE MR images and one clinical feature, achieved an AUC of 0.754 (95% CI, 0.6326 - 0.8745) in the training cohort and an AUC of 0.727 (95% CI, 0.5449 - 0.9097) in the validation cohort (37). Another study, utilizing multiple MRI sequences, included 19 radiomic features and 3 clinical risk factors, predominantly wavelet features except for one original feature. These features were used to predict LVSI preoperatively in patients with stage IB-IIB CC, achieving a C-index of 0.78 and 0.82 (38). While, the integration of combined T1 dynamic contrast-enhanced (DCE-T1) and T2-weighted imaging (T2WI) MRI sequences achieved an AUC of 0.911 for detecting LVSI in cervical cancer (sensitivity = 0.881, specificity = 0.752) (39). The popularity of PET/CT in follow-up visits, combined with 401 PET/CT radiomics features and immunohistochemical markers TNC and COX-2, enabled the construction of predictive models in training and external datasets, achieving AUCs of 0.914 and 0.806, respectively (p < 0.001) (40). Radiomics parameters are characterized by high technical complexity and require further standardization to enhance clinical utility. Our study focuses on incorporating relatively simple and easily obtainable parameters into the model. Looking ahead, integrating additional radiological features could further optimize the preoperative assessment of LVSI.

#### Strengths and Limitations

Considering the inherent bias of retrospective studies, this study has the following limitations. Firstly, Other relevant serum biomarkers and MRI-based radiomics can be utilized for preoperative prediction of LVSI. Secondly, preoperative analysis should be further stratified based on different histological types, levels of cell differentiation, and neoadjuvant therapy. Thirdly, there is no semi-quantitative analysis of LVSI. Studies have shown that diffuse LVSI increases the risk of lymph node metastasis. Semi-quantitative analysis of LVSI may offer a more truthful risk model and guide individualized treatment (41). Therefore, more detailed Semi-quantitative analysis of LVSI is needed, and multi-center prospective randomized clinical trials with larger sample sizes are needed to objectively evaluate LVSI.

## Conclusions

In summary, our nomogram prediction model has demonstrated reliable LVSI predictive performance across different staging systems. Our study highlights the necessity of preoperative MRI assessment for DSI, supporting the extension of this model to preoperative applications.

## Data Availability

The original contributions presented in the study are included in the article/**Supplementary Material**. Further inquiries can be directed to the corresponding authors.
