# Supplementary material for: Nomogram prediction of the lymph-vascular space invasion in cervical cancer: comparison of 2009 and 2018 staging systems
Source: Front Oncol. 2025 Mar 6;15:1505512. doi: 10.3389/fonc.2025.1505512 (PMC11937894; doi:10.3389/fonc.2025.1505512)
Supplement: Supplementary file 1 [file Table1.doc]

**Supplementary Table 1. Characteristics of the 2009 FIGO** **Cohort 1 and 2018 FIGO Cohort 2**

| **Characteristics** | **Total**  **(n = 691)** | **the FIGO 2009 Cohort1 (n = 348)** | **the FIGO 2018 Cohort2  (n = 343)** | **p** |
| --- | --- | --- | --- | --- |
| **age, Mean ± SD** | 48.5 ± 9.3 | 46.4 ± 8.2 | 50.6 ± 9.9 | < 0.001 |
| **FIGO, n (%)** |  |  |  | < 0.001 |
| IB1(cohort1) IB1-IB2(cohort2) | 343 (49.6) | 181 (52) | 162 (47.2) |  |
| IB2(cohort1) IB3(cohort2) | 107 (15.5) | 70 (20.1) | 37 (10.8) |  |
| IIA1(cohort1) IIA1(cohort2) | 92 (13.3) | 53 (15.2) | 39 (11.4) |  |
| IIA2(cohort1) IIA2(cohort2) | 78 (11.3) | 44 (12.6) | 34 (9.9) |  |
| ⅢC(r)(cohort2) | 71 (10.3) | 0 (0) | 71 (20.7) |  |
| **DSI, n (%)** |  |  |  | < 0.001 |
| <1/3 | 251 (36.3) | 160 (46) | 91 (26.5) |  |
| ≥1/3,<2/3 | 219 (31.7) | 148 (42.5) | 71 (20.7) |  |
| ≥2/3 | 221 (32.0) | 40 (11.5) | 181 (52.8) |  |
| **tumor.size, n (%)** |  |  |  | | 0.003 | | --- | |
| <2cm | 239 (34.6) | 142 (40.8) | 97 (28.3) |  |
| ≥2cm,<4cm | 266 (38.5) | 128 (36.8) | 138 (40.2) |  |
| ≥4cm,<5cm | 114 (16.5) | 49 (14.1) | 65 (19) |  |
| ≥5cm | 72 (10.4) | 29 (8.3) | 43 (12.5) |  |
| **pathology, n (%)** |  |  |  | 0.985 |
| Squamous cell carcinoma | 524 (75.8) | 264 (75.9) | 260 (75.8) |  |
| Non Squamous cell carcinoma | 167 (24.2) | 84 (24.1) | 83 (24.2) |  |
| **SCC-Ag (ng/ml), n (%)** |  |  |  | 0.07 |
| <1.5 | 318 (46.0) | 172 (49.4) | 146 (42.6) |  |
| ≥1.5 | 373 (54.0) | 176 (50.6) | 197 (57.4) |  |
| **CA125(U/ml), n (%)** |  |  |  | 0.669 |
| < 35 | 612 (88.6) | 310 (89.1) | 302 (88) |  |
| ≥ 35 | 79 (11.4) | 38 (10.9) | 41 (12) |  |
| **CA199(U/ml), n (%)** |  |  |  |  |
| < 37 | 645 (93.3) | 328 (94.3) | 317 (92.4) | 0.334 |
| ≥ 37 | 46 ( 6.7) | 20 (5.7) | 26 (7.6) |  |
| **NE(%), Mean ± SD** | 58.4 ± 11.0 | 57.2 ± 10.9 | 59.5 ± 10.9 | 0.006 |
| **LY(%), Mean ± SD** | 31.2 ± 9.1 | 31.6 ± 9.0 | 30.9 ± 9.2 | 0.381 |
| **WBC(109/L), Mean ± SD** | 6.3 ± 2.1 | 6.3 ± 2.0 | 6.3 ± 2.2 | 0.861 |
| **PLT(109/L), Mean ± SD** | 256.3 ± 67.3 | 256.7 ± 72.0 | 256.0 ± 62.2 | 0.89 |
| **pelvic.node, n (%)** |  |  |  | < 0.001 |
| Negative | 555 (80.3) | 298 (85.6) | 257 (74.9) |  |
| Positive | 136 (19.7) | 50 (14.4) | 86 (25.1) |  |
| **LDH, Median (IQR)** | 165.1 (143.0, 196.6) | 162.3 (137.2, 198.7) | 166.5 (148.0, 196.0) | | 0.449 |  | | --- | --- | |
| **NLR, Median (IQR)** | 1.8 (1.4, 2.5) | 1.8 (1.4, 2.3) | 1.9 (1.5, 2.6) | 0.069 |
| **Pelvic lymph node (IQR)** | 26.0 (21.0, 33.0) | 25.0 (21.0, 31.0) | 27.0 (21.0, 34.0) | | 0.011 | | --- | |
| **LVSI, n (%)** |  |  |  | < 0.001 |
| Negative | 409 (59.2) | 235 (67.5) | 174 (50.7) |  |
| Positive | 282 (40.8) | 113 (32.5) | 169 (49.3) |  |
