# Supplementary material for: Nomogram prediction of the lymph-vascular space invasion in cervical cancer: comparison of 2009 and 2018 staging systems
Source: Front Oncol. 2025 Mar 6;15:1505512. doi: 10.3389/fonc.2025.1505512 (PMC11937894; doi:10.3389/fonc.2025.1505512)
Supplement: Supplementary file 2 [file Table2.doc]

**Supplementary Table S2. Univariate and multivariate analysis for LVSI in Cohort1**

| **Characteristics** | **crude.OR (95%CI)** | **crude.P value** | **adj.OR (95%CI)** | **adj.P value** |
| --- | --- | --- | --- | --- |
| **DSI** |  |  |  |  |
| <1/3 | 0.15 (0.1~0.24) | <0.001 | 0.67 (0.01~47.45) | 0.854 |
| ≥1/3,<2/3 | 5.94 (3.39~10.41) | <0.001 | 6.75 (3.54~12.89) | <0.001 |
| ≥2/3 | 8.09 (3.73~17.54) | <0.001 | 11.33 (4.5~28.53) | <0.001 |
| **age** | 0.99 (0.96~1.01) | 0.343 | 0.96 (0.92~0.99) | 0.017 |
| **FIGO** |  |  |  |  |
| IB1 | ref |  |  |  |
| IB2 | 1.06 (0.59~1.92) | 0.835 | 0.64 (0.27~1.55) | 0.323 |
| IIA1 | 1.22 (0.64~2.31) | 0.552 | 0.55 (0.23~1.3) | 0.175 |
| IIA2 | 1.02 (0.5~2.06) | 0.967 | 0.58 (0.2~1.63) | 0.3 |
| **tumor.size** |  |  |  |  |
| <2cm | ref |  |  |  |
| ≥2cm,<4cm | 1.76 (1.05~2.95) | 0.032 | 0.7 (0.35~1.41) | 0.32 |
| ≥4cm,<5cm | 1.38 (0.68~2.78) | 0.375 | 0.52 (0.18~1.5) | 0.228 |
| ≥5cm | 1.73 (0.75~4.01) | 0.198 | 0.65 (0.19~2.25) | 0.496 |
| **pathology** |  |  |  |  |
| Squamous cell carcinoma | ref |  |  |  |
| Non Squamous cell carcinoma | 0.18 (0.09~0.38) | <0.001 | 0.17 (0.07~0.39) | <0.001 |
| **SCC-Ag** |  |  |  |  |
| <1.5 | ref |  |  |  |
| ≥1.5 | 2.94 (1.83~4.71) | <0.001 | 2.64 (1.36~5.14) | 0.004 |
| **CA125** |  |  |  |  |
| < 35 | ref |  |  |  |
| ≥ 35 | 1.09 (0.54~2.22) | 0.808 | 1.14 (0.45~2.89) | 0.779 |
| **CA199** |  |  |  |  |
| < 37 | ref |  |  |  |
| ≥ 37 | 1.42 (0.56~3.57) | 0.461 | 1.52 (0.45~5.15) | 0.498 |
| **NE** | 1.01 (0.99~1.03) | 0.451 | 1.01 (0.97~1.05) | 0.575 |
| **WBC** | 0.94 (0.84~1.06) | 0.323 | 0.95 (0.79~1.15) | 0.625 |
| **LY** | 0.99 (0.97~1.02) | 0.599 | 1.01 (0.96~1.07) | 0.582 |
| **PLT** | 1 (1~1) | 0.315 | 1 (0.99~1) | 0.214 |
| **LDH** | 1 (1~1) | 0.006 | 1 (1~1) | 0.057 |
| **NLR** | 1.05 (0.98~1.12) | 0.156 | 1.06 (0.95~1.18) | 0.28 |
